# Drivers of digital transformation adoption: A weight and meta-analysis

**DOI:** 10.1016/j.heliyon.2022.e08911

**Published:** 2022-02-05

**Authors:** Diego Rodrigues Cavalcanti, Tiago Oliveira, Fernando de Oliveira Santini

**Affiliations:** a NOVA Information Management School (NOVA IMS), Universidade Nova de Lisboa, Campus de Campolide, 1070-312 Lisboa, Portugal; b UNISINOS Business School, Universidade do Vale do Rio dos Sinos, Campus de Porto Alegre, 93022-750 Porto Alegre, Brazil

**Keywords:** Digital transformation, Emerging technologies, Digital disruption, Digital innovation, Adoption theories, Individual adoption

## Abstract

The advent of the global pandemic has accelerated the growing need for product and service transformation, highlighting the emerging importance of technology and creating the opportunity to update the digital transformation (DT) domain through empirical-quantitative research. This weight and meta-analysis enabled the synthesis and integration of previous literature on the scope of individual DT adoption, evaluating the state of the art and filling a void on the subject. Athwart 88 studies and 99 datasets by international sources, our results demonstrate that attitude and satisfaction are relevant predictors of behavioral intentions and promising outcomes, including compatibility and personal innovativeness. Behavioral intentions, satisfaction, and habit are the best predictors for DT use. Usefulness and ease of use are critical for DT adoption intention and use, being moderated by individualism, as a cultural factor, human capital, and knowledge-technology, as innovation indicators. We present a conceptual model of promising and best predictors for future research on DT individual adoption.

## Highlights

• An update of digital transformation (DT) through a weight and meta-analysis.
• Contribution to DT literature by surpassing biases and limitations of size estimates.
• Identification of promising and best predictors for further DT adoption research.
• Behavioral intentions, satisfaction, and habit are best predictors for DT use.
• Usefulness and ease of use are pivotal, being moderated by culture and innovation.

## 1 Introduction

The current scenario of high competition and the need for constant innovation makes digital transformation (DT) essential for creating differentiation mechanisms and disruptive business (Jahanmir et al., 2020). Contextualizing the actual society transformed by digital innovation, a report by the world economic forum confirmed the potential of digitalization to generate almost $100 trillion in value for companies and communities in the next decade, accelerating social progress (World Economic Forum, 2020).

Defined as the enhancement of existing products through digitization and digital innovation resources (Vial, 2019), DT is a multidisciplinary theme that encompasses changes in several spheres (Verhoef et al., 2021), such as strategy (Matt et al., 2015), people (Navaridas-Nalda et al., 2020), technology (Pillai et al., 2020), culture (Udo et al., 2016), social and organizational structures (Selander and Jarvenpaa, 2016), affecting the way that companies interact with clients (Jain et al., 2021). However, contrary to what many people think, the most relevant sphere for DT is people, since even if technologies evolve quickly, what really matters is whether people are adopting them (Vial, 2019). For Kane (2019), individuals adopt disruptive technologies more quickly than organizations, which often need more time and flexibility to adapt themselves, making room for further investigation into DT individual adoption.

Only in the last ten years was it possible to identify research in information systems disciplines addressing topics such as digitization, digitalization, and DT (Verhoef et al., 2021), which attests to the lack of interest to investigate advances brought by the introduction of disruptive technologies. Recent literature reviews on DT denoted the use of synonyms such as "digital technology" (Matt et al., 2015), as well as myriad definitions of the term, according to technology type, mainly being focused on organizational impact (Vial, 2019). Few studies can be seen at the micro-level, considering disruptive technology adoption through an individual's vision (Meske and Junglas, 2020; Nadkarni and Prügl, 2021), as Verhoef et al. (2021) highlighted greater attention to studies on the adoption of digital technologies and their impact on business only.

As DT brings digital culture premised on giving more power to individuals, increasing its importance in the transformative equation (Guy, 2019), a gap was identified in the literature on individual adoption. Previous research presented conflicts and inconsistencies in results (Blut et al., 2021), considering the wide variation between the effects of main predictors and the outcomes for individual adoption of disruptive technologies, which contradict the conclusions of main adoption models. Some studies found a negative impact for ease of use in behavioral intentions (Çera et al., 2020; Vimalkumar et al., 2021), while others present a strong positive relationship among the same constructs (Khaksar et al., 2021; Pillai et al., 2020). Similarly, for disruptive technology adoption such as DT, a negative relation was found between usefulness and behavioral intentions (Nastjuk et al., 2020; Sobti, 2019), as opposed to the original model's prediction (Venkatesh et al., 2003, 2012), in addition to other studies obtained (Cabrera-Sá nchez et al., 2021; Kabir, 2020). Even for other outcomes such as use behavior, a given study shows a negative relationship with facilitating conditions (*r* = -.04; Chopdar et al., 2018), while other studies adduce a weak impact (*r* = .16; Alam et al., 2021; Vimalkumar et al., 2021), and yet another one accounts for the strong impact of the ratio (*r* = .52; Netshirando et al., 2020). For Blut et al. (2021), these inconsistencies can be attributed to the non-complete application of the theoretical models, the use of a small sample, or the disregard of variations arising from the different contexts or specificities of the investigated technology.

The presented scenario creates opportunities for investigating the DT phenomenon through the lens of adoption theory (Venkatesh et al., 2003), focusing on individuals to help resolve inconsistencies in previous research, clarify the main DT predictors, and answer why people accept disruptive technologies. This research fills this lacuna by conducting a weight and meta-analysis to review existing studies and systematize the empirical results (Cram et al., 2019), bringing accurate and reliable conclusions to disruptive technologies adoption such as DT. Thus, the objectives are two-fold: 1) providing an overview of the pivotal factors for DT adoption under individual focus, proposing a specific and reliable acceptance model; 2) contributing to DT literature by surpassing biases and limitations of size estimates in previous research (Cram et al., 2019), and identifying potential and best predictors for further investigation (Baptista and Oliveira, 2019).

This study is organized as follows. Initially, we broach the research methodology with the problem definition, expound on the studies' selection or rejection criteria, data extraction, and merge the variable process. Then we delve into a descriptive analysis followed by the weight and meta-analysis results and moderation analysis. After, we discuss the findings and implications for theory and practice, and finally, elucidate on the limitations, future avenues for research, and outline the conclusions.

## 2 Digital transformation and technology adoption models

Disruptive technologies have transformed the way companies and individuals interact, making the provision of services and consumption more flexible, evoking the need for continuous innovation. Organizations need to understand the transformative technology adoption process better, as well as the intention, acceptance, and use of those technologies by users to survive in this increasingly dynamic and competitive environment (Jahanmir et al., 2020). For Carroll (2020), technological changes are analogous to transformation, which in turn deal with fundamental changes for carrying out a differentiated job, given market pressures or new opportunities.

In the mid-1990s, the emergence of the commercial internet gave rise to new market and business models, introducing the first ideas about DT and highlighting the central role of IS in value creation processes (Meske and Junglas, 2020). Since then, there has been little evolution in the concept of DT (Vial, 2019), and currently, there is still no fully accepted definition (Mergel et al., 2019). As a multidisciplinary approach, DT is activated by a corporate trigger, as a response by organizations to adopting digital technologies, changing the individual behavior of customers and consumers, and increasing digital competition (Verhoef et al., 2021). DT translates into better interactions between suppliers, customers, and competitors (Singh and Hess, 2017).

Highlighting the lack of studies on the individual human aspects of the topic, Kane (2019) presented his differentiated view of digital transformation with the "technological fallacy," defending the human and organizational aspects as more essential elements than the technological ones. For him, transformative technologies involve changes in how work is performed, making human factors a determinant of organizational digital maturity. In the same direction, Carroll (2020) sought to normalize DT in practice, strengthening the argument that the success of DT does not depend on digital technologies but on the individuals who adopt and use the technology, cultural changes, and organizational processes.

The IS research field, in the last decades, has made great efforts to understand why individuals accept and adopt transformative technologies and how quickly they are used (Davis, 1989; Venkatesh et al., 2003), given that disruptive technologies can raise the level of competition, replacing the old existing pattern (Schmidthuber et al., 2020). Nevertheless, studies on technological adoption can be carried out under different focuses such as individual and organizational (Vimalkumar et al., 2021), in addition to varied theories and models (Rahi et al., 2019), such as the Diffusion Theory of Innovation (DOI) (Rogers et al., 2019), Theory of Planned Behavior (TPB) (Ajzen, 2011), Theory of Rational Action (TRA) (Fishbein and Ajzen, 1975), Social Cognitive Theory (SCT) (Bandura, 1986) and Motivational Model (MM) (Vallerand, 1997), being the most dominant (Mariani et al., 2021) the Technology Acceptance Model (TAM) (Davis, 1989), and the Unified Theory of Acceptance and Use of Technology (UTAUT) (Venkatesh et al., 2003).

A precursor model created to predict attitudes and understand individual behavior in the acceptance of new technologies, the TAM is focused on two constructs considered fundamental to other models, namely, perceived ease of use and perceived usefulness. Since its original proposal, TAM has been evolved to new versions. In 2003, Venkatesh et al. (2003) consolidated different constructs, proposing the UTAUT. After comparing eight adoption models, UTAUT finds that effort expectation, performance expectation, social influence, and facilitating conditions significantly impact the user's intention to adopt technology. Around 2012, UTAUT2 was proposed as the most recent version of the theory applied to the individual consumer and capable of explaining approximately 74 percent of the variations in technology adoption and use from the individual's view (Venkatesh et al., 2012).

Given the relevance of different technologies over existing models, past research has assessed the adoption of different types of disruptive technologies with a transformative focus, such as the autonomous vehicles (Manfreda et al., 2021), internet of things (Ben Arfi et al., 2021b), artificial intelligence (Pillai et al., 2020), blockchain (Queiroz and Fosso Wamba, 2019), Voice-based digital assistants (Vimalkumar et al., 2021), Digital payment (Balakrishnan and Shuib, 2021), mobile payment (Patil et al., 2020), mobile health applications (Alam et al., 2020), Digital Personal Data Stores (Mariani et al., 2021), On-Demand Service Platforms (Delgosha and Hajiheydari, 2020), Business Intelligence and Analytics (Jaklič et al., 2018), social assistive technology (Khaksar et al., 2021), virtual reality (Kunz and Santomier, 2019), besides others considering also varied organizational configurations, as government (Hujran et al., 2020), hospitals (Rahman et al., 2016), schools (Guggemos and Seufert, 2021), retail stores (Pillai et al., 2020) and banks (Hu et al., 2019).

With the context presented and understanding that digital transformation is a broad concept capable of being split into three evolutionary stages (e.g., digitization, digitalization, and digital transformation) (Verhoef et al., 2021), we define individual adoption of DT as the degree to which disruptive and transformative technologies are adopted and/or accepted by individuals, whether employees, consumers, customers or citizens, after an improvement event or development of a new product, process or innovation. The main idea about analyzing DT adoption at an individual level is to examine the variables that influence the individual's intention and the respective choices in favor of accepting or rejecting transformative digital technology. Using as an example of DT individual adoption, the analysis of a client from some customer service, which started from face-to-face assistance going to the use of chatbots and artificial intelligence in an online channel, we understand that DT affects organizations and society, changing the interaction between people and services (Carroll, 2020).

## 3 Research methodology

### 3.1 Criteria

The initial step was to define the concept and criteria to be adopted in this search and summarize the research published on DT adoption at an individual level. The first academic definitions for DT appeared in Bowersox et al.'s works in 2005 as a business reinvention process for supply chain management (Schallmo et al., 2017). The subject's importance gave rise to a pervasive variability of definitions (Kraus et al., 2021), and the absence of a common definition initiated the use of synonyms such as digitalization, digitization, digital disruption, and others (Mergel et al., 2019). With a broader definition of DT, it becomes possible to identify studies on the individual, moving away from a definition centered on the organization (Vial, 2019) to something that fits social and individual contexts of transformative technology, closer to the concept of digitalization (Mergel et al., 2019). Thus, an embracing definition was adopted as a process to improve an entity, producing significant changes in its assets through the use of technology and computing, combined with communication and connectivity (Vial, 2019).

Based on previous studies (Mergel et al., 2019; Scott et al., 2019; Verhoef et al., 2021; Vial, 2019), the appropriate keywords for the research were selected, namely DT, transformation, digitalization, digitization, emerging technology, digital disruption, and digital innovation. For adoption theory we used, adoption, intention to adopt, individual adoption, adoption intention, use intention, intention to use, and behavioral intention. All possible combinations, with the logical operators 'AND' and 'OR' connecting the presented keywords for the query (Baptista and Oliveira, 2019), were applied in different research databases: Scopus, ACM digital library, EBSCO, Emerald, Taylor & Francis, Springer, Web of Science, Science Direct, JSTOR, and Google Scholar. For electronic database searches, two themes were specified. The boolean search strategy was utilized using the term "and" (Gerow et al., 2014): DT subject ("digital transformation" or "digitalization" or "digitization" or "emerging technology" or " digital disruption" or "digital innovation"), and adoption theory ("adoption" or "intention to adopt" or "individual adoption" or "adoption intention" or "use intention" or "intention to use" or "behavioral intention").

The initial search considered all publication types (journals, articles, reviews, conferences, and books) and resulted in more than 1,861 publications between 2014 and the beginning of 2021, requiring refinement. We emphasize that no cut-off point was used and that quantitative studies correlating DT and individual technology adoption in the databases were only found from 2014 onwards. Even so, only around 2018, a considerable evolution was noted in the number of empirical studies related to the topic and has apparently had an evolutionary profile since then.

**Figure 1:**
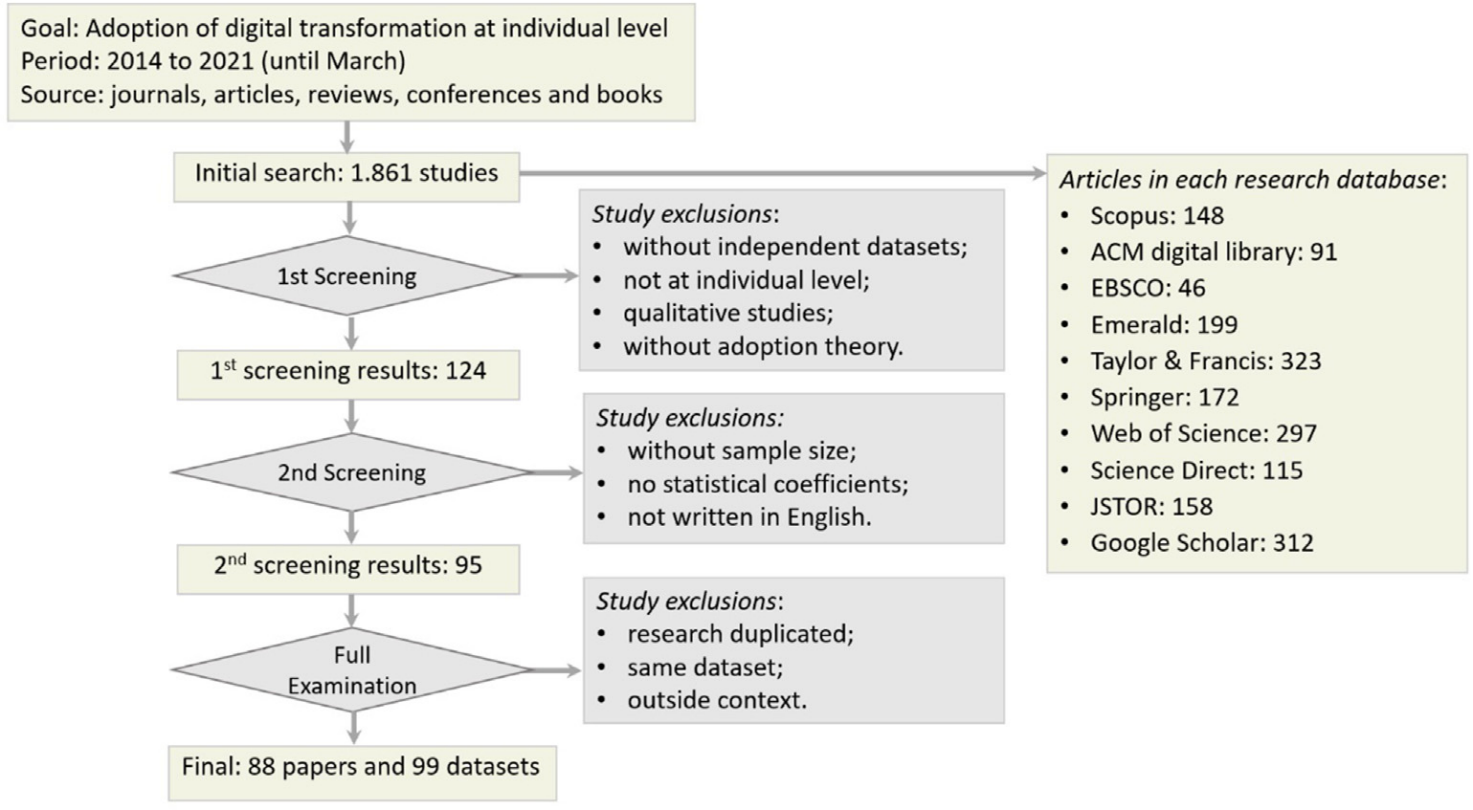
Studies' selection process.

The second stage involved selecting studies according to general criteria: focus on individual-level analysis, empirical nature and quantitative results, technology adoption theory context, and independent datasets. The result was 124 publications, which were then submitted to new criteria necessary for the meta and weight analysis, as follows: report sample size, statistical coefficients, and written in the English language. The resulting 95 articles also underwent a thorough examination to avoid bias problems (Franque et al., 2020), excluding duplicated publications, same respondents' datasets, studies not related to DT adoption, or those that only cited the selected keywords without a transformative context. Research with multiple independent databases was included, for instance, the studies of (Thakurta et al., 2020) (Scott et al., 2019) (Queiroz and Fosso Wamba, 2019) (Chopdar et al., 2018) (El-Masri and Tarhini, 2017) (Kummer et al., 2017), and (Udo et al., 2016) with two datasets, and (Taghizadeh et al., 2021) with five, resulting in 88 articles (85 from journals and three conference papers) and 99 useful datasets, as depicted in Figure 1.

The sample is adequate and allows results generalization (Santini et al., 2019) when compared to recent studies published in top journals, such as (Naranjo Zolotov et al., 2018) with 60 studies (Baptista and Oliveira, 2019), with 54 studies (Franque et al., 2020), with 115 studies, and (Jadil et al., 2021) with 127 studies.

The extraction data process from the 88 selected studies initially involved collecting primary data such as study name, author, place and year of publication, methodology and theories used, geographic origin, quantity and type of sample, and technology. Then, quantitative statistical information on the relations between variables was collected. Names and concepts were merged to assess the relationships between variables and increase meta-analysis precision (Jadil et al., 2021), as similarities were found in original nomenclatures between variables (Blut et al., 2021). The aggregating and re-organizing process followed Venkatesh et al. (2003) approach to allow construct unification. However, given the pronounced variability of technology topics used by authors, it was decided to keep similar but non-identical constructs, such as relative advantage and usefulness; facilitating conditions, perceived behavioral control and compatibility; ease of use, and complexity, as described in Table 1, which provides all codifications and redefinitions of the variables used.

**Table 1:** Coding and constructs definition.

Construct	Definition	Original names collected in the dataset
Attitude	Positive or negative feelings about the performance of an individual intended behavior (Fishbein and Ajzen, 1975)	attitude, attitudes content, attitudes technology, attitude toward technology use, Attitude Toward Using, Attitude Toward Use, Attitude Toward transition, Users Attitude, Overall attitude
Behavioral Intentions	The strength of the individual intention to perform a certain behavior (Fishbein and Ajzen, 1975)	acceptance intentions, adoption, adoption intentions, behavior intentions, behavioral intentions to use, continuance intentions, intentions, intentions to actively support, intentions to adopt, intentions to use, use intentions, usage intentions, customers intentions, switching intent, switching intention, purchase intentions, interest to use, routine use intentions, intentions to transition
Compatibility	The degree of consistency and adequacy of an innovation to the needs, experiences, and values of the adopters (Rogers et al., 2019)	perceived compatibility
Confirmation	Indicates the validation of the individual's basic expectations after interaction with a certain context or system use (Bhattacherjee, 2001)	-
Complexity	Individual perception about the difficulty of understanding and using an innovation (Rogers et al., 2019)	-
Ease of Use	The degree of ease in using a given technology (Venkatesh et al., 2003)	effort expectancy, effort expectation, perceived ease of use, pre-adoption effort expectancy
Facilitating Conditions	Perception about support and available resources for conducting a behavior (Venkatesh et al., 2003)	perceived facilitating condition
Habit	Represents the execution of automatic individual behavior after learning (Venkatesh et al., 2012)	incumbent system habit
Hedonic Motivation	Fun or pleasure from the use of technology (Venkatesh et al., 2012)	enjoyment, perceived enjoyment
Innovativeness of IT	Individuals tendency to adopt new technology much earlier and more frequently than others (Rogers et al., 2019)	customer innovativeness, innovativeness, user innovation
Perceived Behavioural Control	Perceived control when performing a certain behavior (Orbell et al., 1997)	perceived behavior control
Perceived Costs	Refers to general transaction costs, involving time and effort perceived by the individual (Featherman and Pavlou, 2003)	-
Perceived Value	Degree of association between value and usefulness perceived by the individual, in terms of time and effort invested (Dodds et al., 1991)	-
Personal Innovativeness	Individual characteristic that denotes the willingness to try new technologies (Agarwal and Karahanna, 2000)	perceived personal innovativeness
Price Value	Perception regarding the benefit of something, versus the cost to use it (Dodds et al., 1991)	price, price evaluation, price benefit
Privacy Risk	Possible loss of control over personal information, which is used without the owner's permission or knowledge (Featherman and Pavlou, 2003)	privacy concerns, privacy, perceived privacy risk, risk
Relative Advantage	Individuals' perception that a recent innovation is better than the previous one (Rogers et al., 2019)	perceived relative advantage
Risk	Perception about uncertainties after expectations about adverse results from system use (Fu et al., 2006)	perceived risk
Satisfaction	Affective emotional reaction after an experience with a certain technology (Bhattacherjee, 2001)	user satisfaction, perceived satisfaction
Self-Efficacy	Individuals' belief about the ability to perform specific tasks using a system (Venkatesh, 2000)	computer self-efficacy, post-adoption self-efficacy
Self-Quarantine	Restriction of people who are presumed to have been exposed to a contagious disease (Alam et al., 2021)	-
Social Influence	User perception of how important people believe that he should use some technology (Venkatesh et al., 2003)	subjective norm, subjective social norm, perceived social influence
Trust	Perception of reliability, which involves a set of beliefs related to integrity, benevolence, and competence (Gefen et al., 2003)	consumer trust, trust belief, users trust, trust of stakeholders, trust in the system, perceived trust, trust concerns
Use Behavior	Current use of the system from the point of view of technology acceptance (Davis, 1989)	actual usage, actual usage behavior, actual behavior, adoption, technology adoption, use behavior, usage, intentions to continue usage, continuance usage intentions, usage behavior, objective use, service use
Usefulness	The degree to which some technology provides benefits to users when performing activities (Venkatesh et al., 2003)	expected performance, perceived usefulness, performance expectancy, post-adoption perceived usefulness, pre-adoption performance expectancy

### 3.2 Weight and meta-analysis

As a quantitative technique for analyzing a large number of empirical publications (Jadil et al., 2021), meta-analysis is a theoretical extension tool for the evaluation of models' evolution (Blut et al., 2021), allowing effect size comparison between studies (Geyskens et al., 2009). Meta-analysis makes it possible to generalize results after investigating the set of conclusions obtained from studies adopting different methods, samples, and techniques (Borenstein et al., 2009). Meta-analysis was selected since it offers several benefits (Santini et al., 2019) to examine technology adoption models applied to DT. First, it allows testing assumptions not previously evaluated, quantifying moderating influences, adding and excluding constructs, evaluating and revising variables to expand existing models (Blut et al., 2021). Second, it gives an overview of the constructs in a given research topic, exploring the relationships between predictors and achieved results (Cram et al., 2019). Blut et al. (2021) explained that the last benefit exists in several examples of meta-analysis used to review models in the technology adoption context, such as the TAM, IS success model, TRA and TPB, expectation theory ECM), and the UTAUT.

We used random effects to estimate the statistical summary effect and calculate the studies' variability, which is convergent with this research, preventing extensive studies from dominating the analysis and assuming that effect sizes vary between studies (Borenstein et al., 2009). The meta-analysis covers the most frequently used relations, which occurred three or more times in the 88 selected publications. The meta and weight analysis table presents two initial columns containing the relationship between dependent and independent constructs. The "Sample" column shows the cumulative samples, "Correlation (*r)*" represents the average of correlation coefficients corrected by sample size, "Confidence interval" brings up the lower and upper limit of the 95% confidence interval (Baptista and Oliveira, 2019). For the FSN, the Rosenthal method was used to verify the number of articles required for the result to be false (Santini et al., 2016), serving to assess relation robustness (Borenstein et al., 2009) and publication bias (Blut et al., 2021).

The Q and I^2^ tests were used to assess the significance of heterogeneity among studies (p < 0.05). The I^2^ index varies from 0 to 100%, as 25% represents low and 75% shows high heterogeneity (Borenstein et al., 2009). After the heterogeneity test, high levels were found in the publications of our dataset since only seven of the 48 relationships are below 90%. The asymmetry test was then performed using Egger regression, which was significant for asymmetry (p-value>0.10 for 38 of 48 relationships). However, no evidence was found indicating publication bias in the dataset, but just a high level of heterogeneity, convergent with previous research (Naranjo Zolotov et al., 2018).

The weight analysis was obtained by the ratio of significant variable frequency to the number of tests for the same variable. This technique estimates predictor importance and relationship intensity between constructs (Jeyaraj et al., 2006). The influence of independent variables on dependent ones was analyzed, considering relations examined three or more times (Baptista and Oliveira, 2019; Franque et al., 2020), totalizing 48 relationships. Predictors were classified into (Jeyaraj et al., 2006): (I) "Best Predictors," for relationships among variables explored five or more times in the dataset and weighing more than 0.80 (80%); and (II) "Promising Predictors," for relations explored four or fewer times in the dataset, and weighing equal to 1 (100%). Weight 1 indicates significance for all relations in the dataset, while weight 0 shows insignificance (Jeyaraj et al., 2006).

### 3.3 Moderators

Regarding the moderators' assessment in adoption models, after revision of the UTAUT model and possible extensions identified in the literature, Venkatesh et al. (2016) cite the need for more studies that show the context of the theoretical application of adoption models. Further studies on technology adoption encompassing different moderators are needed to understand the variability of organizations, cultures, and various technologies as contextual predictors of UTAUT (Blut et al., 2021). We selected gross domestic product (GDP), cultural factors, and innovation factors as moderators. Economic differences such as the income level between countries is also a relevant factor since the Tallon & Kraemer framework relates different use and adoption of technology according to a country's economic development (Švarc et al., 2020). As societies with higher income levels have a greater economic capacity and ample access to resources, which positively influences disruptive technology adoption (Kim and Peterson, 2017), a stronger relationship between individual adoption of DT is expected in countries with higher GDP.

Given the importance of the innovation index for DT adoption, individual indicators that build the index were measured. The individual indicators developed by the world intellectual property organization (WIPO, 2020) are institutions (like political, regulatory, and business environment subdimensions), human capital and research (including education, tertiary education, and research & development – R&D), infrastructure (with ICTs, general infrastructure, and ecological sustainability), market sophistication (like credit, investment, trade, competition, and market scale), business sophistication (with knowledge workers, innovation linkages, and knowledge absorption), knowledge and technology outputs (with creation, impact, and diffusion of knowledge), and finally creative outputs (like intangible assets, creative good-services, and online creativity). A strong relation among individual adoption of DT is expected in countries with a higher innovation index since individuals from nations with high innovative rates tend to have greater skills, structure, and competencies (Santini et al., 2019) to adopt new technologies.

As cultural variability can influence individual behavior, explaining technology use in different cultures (Peña-García et al., 2020; Srite and Karahanna, 2006), the original Hofstede's model with four factors for cross-country comparisons was used: power distance, individualism, masculinity, and uncertainty avoidance (Hofstede, 2001).

A stronger relationship between individual adoption of DT in countries with high individualism and masculinity is proposed, considering the focus on advancement, goals, competitiveness, and performance values (Hofstede, 2001; Srite and Karahanna, 2006). A weaker relationship is expected between adoption and disruptive technologies in countries with greater power distances and uncertainty avoidance, given that societies with characteristics of centralization in organizational decisionmaking, process formalization, and resistance to change (Hofstede, 2001) present difficulties and adverse factors to disruptive technology adoption (Chopdar et al., 2018).

## 4 Results

### 4.1 Descriptive analysis

Of the 88 studies found, 85 are articles and three conference papers, with a total of 442 useful relationships (independent-dependent variable) under the criteria of meta-analysis and for weight analysis input. Anent the researched period, more than 88% (78 publications) refer to the last four years, with nine investigations from 2018, 12 from 2019, 34 from 2020, and 23 publications from 2021 (considering only the first three months of this year), which shows a growing interest in the theme, as shown in Table 2. Respondents' data by country and year (Table 3) confirms the growth trend of DT research from 2016, reaching 13,733 participants in 2020 and 9,046 in 2021, considering only the first three months.

**Table 2:** Research studies by source and year.

N	Source	2014	2015	2016	2017	2018	2019	2020	2021∗	Total
1	African Journal of Science, Technology, Innovation and Development							1		1
2	Asian Economic and Financial Review							1		1
3	Asian Journal of Technology Innovation							1		1
4	Behaviour & Information Technology							2	2	4
5	Computer Standards & Interfaces					1				1
6	Computers in Human Behavior	1		1		3		1	2	8
7	Decision Support Systems	1								1
8	Educational Technology & Society								1	1
9	Educational Technology Research and Development			1	2					3
10	Electronic Commerce Research and Applications						1			1
11	Government Information Quarterly						1		1	2
12	Health and Technology							1		1
13	IEEE Access								1	1
14	Information & Management				1					1
15	Information Systems Management							1		1
16	Information Technology & People								2	2
17	Information Technology for Development							1		1
18	International Conference on Computational Intelligence in Data Science (ICCIDS)					1				1
19	International Conference on Distance Education and Learning (ICDEL)							1		1
20	International Conference on IT Systems and Innovation (ICITSI)							1		1
21	International Journal of Human-Computer Interaction							1		1
22	International Journal of Information Management			1		1	4	3		9
23	International Journal of Medical Informatics					1				1
24	International Journal of Scientific &Technology Research							1		1
25	International Journal of Technology Management							1		1
26	Journal of Advances in Management Research						1			1
27	Journal of Biomedical Informatics								1	1
28	Journal of Business Ethics			1						1
29	Journal of Business Research							2	1	3
30	Journal of Computer Information Systems							1		1
31	Journal of Global Operations and Strategic Sourcing								1	1
32	Journal of Innovation & Knowledge							1		1
33	Journal of Retailing and Consumer Services							1		1
34	Journal of Systems and Information Technology							1		1
35	Machine Learning with Applications								1	1
36	Sport, Business and Management: an international journal							1		1
37	Sustainability							2		2
38	Technological Forecasting and Social Change					1	2	3	6	12
39	Technology in Society		1				1	2	2	6
40	Telecommunications Policy							1		1
41	Telematics and Informatics						1	1	1	3
42	The Journal of Academic Librarianship								1	1
43	Tourism Management					1				1
44	Transforming Government: People, Process and Policy							1		1
45	Transportation Research Part A						1			1
TOTAL		2	1	4	3	9	12	34	23	88

Note: ∗ Only the first three months of the year considered.

**Table 3:** Sample by country and year (ordered by country name).

Country	Year								*Total*
	2014	2015	2016	2017	2018	2019	2020	2021∗	
Albania							380		380
Australia				246				302	548
Austria							670		670
Bangladesh							400	936	1,336
Belgium							202		202
**China**				292	284	387	827	750	2,540
France							316	535	851
Poland			66						66
**Germany**				243		1,067	828	316	2,454
**India**			331		478	1,767	3,615	1,570	7,761
Iran							582	641	1,223
Italy					51				51
Jordan							302	400	702
Malaysia								809	809
Netherlands							624		624
Oman								265	265
Pakistan						398	380	307	1,085
Portugal							141		141
Qatar				418					418
Romania								206	206
Serbia							502		502
Singapore			592				163		755
Slovenia					195	382			577
South Africa							389		389
South Korea						153			153
Spain					256		681	740	1,677
Switzerland								212	212
Taiwan		402			370			285	1,057
Thailand						382			382
Turkey							234		234
UK						398	523	214	1,135
**USA**	530		545	389	934	510	815	558	4,281
Vietnam							1,159		1,159
*Total per Year*	530	402	1,534	1,588	2,568	5,444	13,733	9,046	34,845

Note: highlighted countries have more influence (total sample >2,000 people). ∗Considered only the first three months of the year.

The source analysis reveals that five journals are responsible for almost 45% of the relevant publications (Technological Forecasting and Social Change, International Journal of Information Management, Computers in Human Behavior, Technology in Society, and Behavior & Information Technology), with four or more papers each. Considering the 85 scientific journals used, the impact assessment index (ranking) shows that 75% are in the first quartile, while 17% are in Q2 and 8% in Q3, as Table 2 denotes. The three most used models were TAM (69% - 61 publications), UTAUT (45% - 40 studies), and UTAUT2 (17% - 15 studies), as identified in Table 4.

**Table 4:** Studies used in this research.

ID	Reference	Model	Country	Source	Sample
1	(Navaridas-Nalda et al., 2020)	TAM	Spain	JA	142
2	(Jain et al., 2021)	TRA, TBS, TBRA	India	JA	487
3	(Liébana-Cabanillas et al., 2020b, Liébana-Cabanillas et al., 2020a)	TAM, UTAUT	Spain	JA	539
4	(Guggemos and Seufert, 2021)	TPB	Switzerland	JA	212
5	(Manfreda et al., 2021)	TAM, UTAUT	Slovenia	JA	382
6	(Jahanmir et al., 2020)	TAM, UTAUT	Portugal	JA	141
7	(Ben Arfi et al., 2021a)	UTAUT	France	JA	268
8	(Cabrera-Sá nchez et al., 2021)	UTAUT2	Spain	JA	740
9	(Nastjuk et al., 2020)	TAM	Germany	JA	316
10	(Baudier et al., 2020)	UTAUT2, TAM2	France	JA	316
11	(Pillai et al., 2020)	TAM, TRI	India	JA	1,250
12	(M. Z. Alam et al., 2020)	UTAUT2	Bangladesh	JA	400
13	(Rahman et al., 2016)	TPB, TAM, UTAUT	USA	JA	314
14	(H. Li et al., 2014)	TAM, UTAUT	USA	JA	192
15	(Huang and Chueh, 2020)	TAM, UTAUT	China	JA	258
16	(Chandra et al., 2020)	TAM, UTAUT	Singapore	JA	163
17	(Jetter et al., 2018)	TAM	UK and Italy	JA	51
18	(Yan et al., 2021)	TAM, UTAUT	China	JA	397
19	(Z. Hu et al., 2019)	TAM	China	JA	387
20	(Meske and Junglas, 2020)	TAM, TPB	Germany	JA	149
21	(Khoa, 2020)	TAM	Vietnam	CP	918
22	(Schikofsky et al., 2020)	TAM	Germany	JA	1,067
23	(Chakraborty et al., 2020)	TAM	India	JA	253
24	(Chakraborty et al., 2021)	TAM, TPB	India	JA	146
25	(Scott et al., 2019)	TAM	UK and USA∗	JA	201
26	(S. C. Chen et al., 2018)	TAM, ECM, DOI	Taiwan	JA	370
27	(Y. Chen et al., 2018)	TAM, ELM	China	JA	284
28	(Rodger, 2014)	TAM	USA	JA	338
29	(Rahi et al., 2019)	UTAUT	Pakistan	JA	398
30	(Queiroz and Fosso Wamba, 2019)	TAM, UTAUT	India and USA∗	JA	738
31	(Kamolsook et al., 2019)	UTAUT	Thailand	JA	382
32	(Schmidthuber et al., 2020)	TAM, UTAUT	Austria	JA	670
33	(Chopdar et al., 2018)	UTAUT2	India and USA∗	JA	366
34	(Lau et al., 2021)	TAM	Malaysia	JA	330
35	(Bölen, 2020)	TAM, DOI	Turkey	JA	234
36	(Martínez-Caro et al., 2018)	TAM, D&M	Spain	JA	256
37	(Pillet and Carillo, 2016)	UTAUT, TAM	France and Poland	JA	66
38	(So et al., 2018)	TPB, TAM, UTAUT2	USA	JA	519
39	(Liébana-Cabanillas et al., 2020b, Liébana-Cabanillas et al., 2020a)	TAM, UTAUT	India	JA	206
40	(Pham and Ho, 2015)	TAM, DOI	Taiwan	JA	402
41	(Aswani et al., 2018)	UTAUT2	India	CP	257
42	(Hao et al., 2017)	TAM, UTAUT	China	JA	292
43	(Teo et al., 2016)	TPB, UTAUT	Singapore	JA	592
44	(El-Masri and Tarhini, 2017)	TAM, UTAUT2	USA and Qatar∗	JA	807
45	(Lin et al., 2021)	TAM	Taiwan	JA	285
46	(Udo et al., 2016)	UTAUT, NAM	India and USA∗	JA	562
47	(Kummer et al., 2017)	TAM, UTAUT	Australia and Germany∗	JA	489
48	(Balakrishnan and Shuib, 2021)	UTAUT, TRI	Malaysia	JA	258
49	(Nikou et al., 2020)	TAM, UTAUT	Netherlands	JA	624
50	(M. M. D. Alam et al., 2021)	UTAUT2	Bangladesh	JA	434
51	(Park et al., 2019)	TAM	South Korea	JA	153
52	(Vimalkumar et al., 2021)	UTAUT2	India	JA	252
53	(Gupta et al., 2020)	UTAUT, ECM	India	JA	716
54	(A. Pal et al., 2020)	UTAUT, ECM	India	JA	298
55	(Patil et al., 2020)	TAM, UTAUT	India	JA	491
56	(Mariani et al., 2021)	TAM	UK	JA	214
57	(Rafique et al., 2021)	TAM, ECM	Pakistan	JA	307
58	(Ray et al., 2019)	TAM, SCT	India	JA	513
59	(Milanović et al., 2020)	UTAUT	Serbia	JA	502
60	(Saheb, 2020)	TAM, DOI	Iran	JA	582
61	(Kunz and Santomier, 2019)	UTAUT2	Germany	JA	570
62	(Kar et al., 2021)	TAM, UTAUT	India	JA	685
63	(Kabir, 2020)	TAM, SDT	Bangladesh	JA	215
64	(G. Hu et al., 2020)	TAM, UTAUT	Pakistan	JA	380
65	(Albashrawi and Motiwalla, 2020)	UTAUT, D&M	USA	JA	472
66	(Hajiheydari et al., 2021)	TAM, UTAUT	Iran	JA	427
67	(D. Pal and Patra, 2021)	TAM, TTF	India	JA	232
68	(Nguyen et al., 2020)	UTAUT2	Vietnam	JA	241
69	(Meng et al., 2019)	TAM, TTF	India	JA	270
70	(Ben Arfi et al., 2021b)	UTAUT	France	JA	267
71	(Delgosha and Hajiheydari, 2020)	TAM, UTAUT	UK	JA	523
72	(Johnson et al., 2018)	TAM, DOI	USA	JA	270
73	(Sun et al., 2020)	TAM, UTAUT	China	JA	401
74	(Jaklič et al., 2018)	TAM, UTAUT	Slovenia	JA	195
75	(Thakurta et al., 2020)	TAM, UTAUT	Germany and India∗	JA	278
76	(Alarabiat et al., 2021)	TAM, UTAUT, TPB	Jordan	JA	400
77	(Çera et al., 2020)	TAM, UTAUT2	Albania	JA	380
78	(Sobti, 2019)	UTAUT	India	JA	640
79	(Khan et al., 2020)	TAM, SCT	China	JA	353
80	(Mamonov and Benbunan-Fich, 2020)	TAM, UTAUT	USA	JA	558
81	(P. C. Li et al., 2020)	UTAUT, TTF	China	CP	168
82	(He et al., 2020)	UTAUT, TPB	Belgium	JA	202
83	(Netshirando et al., 2020)	TAM, UTAUT2	South Africa	JA	389
84	(Khaksar et al., 2021)	TAM, DOI	Australia	JA	302
85	(Taghizadeh et al., 2021)	UTAUT, DOI, ECM	Bangladesh, Malaysia, Oman, Romania, and Iran∗∗	JA	1,193
86	(Aldossari and Sidorova, 2020)	UTAUT2	USA	JA	343
87	(Hujran et al., 2020)	TAM, UTAUT2, TPB	Jordan	JA	302
88	(El-Haddadeh et al., 2019)	TAM, UTAUT	UK	JA	313

Notes: ∗Studies with two subsamples; ∗∗ Study with five subsamples; JA = Journal articles; CP = Conference proceeding; D&M – Delone and Mclean IS success model; DOI - diffusion of innovation; ECM - expectation confirmation model; ELM - elaboration likelihood model; NAM - norm activation model; SCT - social cognitive theory; SDT - self-determination theory; TAM - technology acceptance model; TBRA - technology-based reasoned action; TBS - technology-based services model; TPB - theory of planned behavior; TRA - theory of reasoned action; TRI - technology readiness index; TTF - task technology fit; UTAUT - unified theory of acceptance and use of technology.

From the 99 datasets, 34,845 respondents were tabulated in total, briefly categorized into groups, according to Table 5. Mobile technologies showed the most respondents (20,041 respondents - 58% of the total), representing its importance for DT as a reference technology, including banking, health, learning, and smartphone payment applications.

**Table 5:** Respondents grouped by technology.

Technology Type	Respondents	(%)
Blockchain	953	3%
Healthcare Technology	1,332	4%
Social Technology	1,671	5%
Educational Technology	1,749	5%
Internet of things	2,279	6%
Artificial Intelligence	3,224	9%
Digital services-systems	3,596	10%
Mobile	20,041	58%
***Total***	*34,845*	

**Figure 2:**
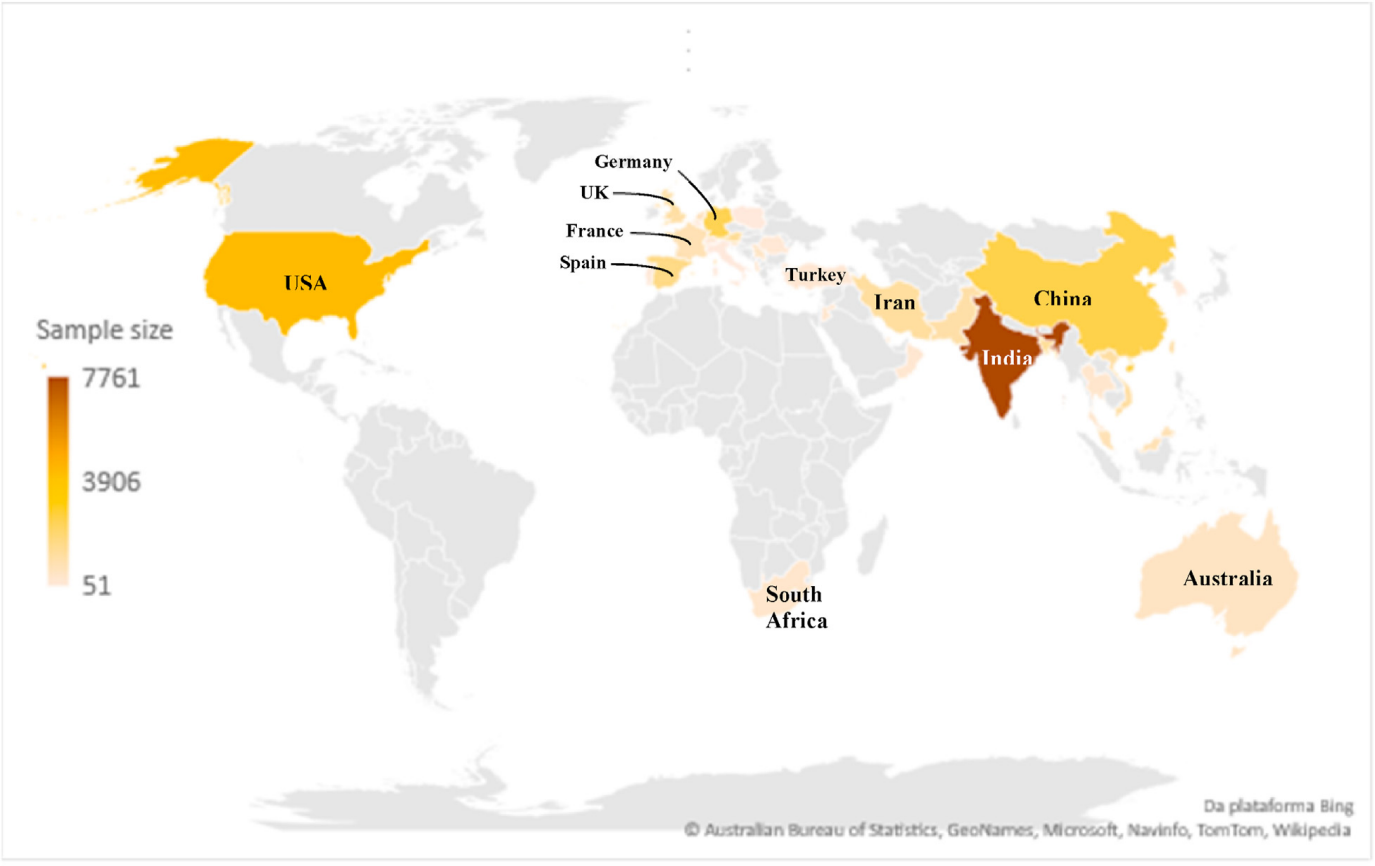
Respondents' distribution by country.

Covering 33 countries, respondents' data by country (Table 3) portrays four countries with more than 2,000 people, responsible for 48% of the total. India has the largest sample size (7,761 individuals, with 22% of total), followed by the United States (4,281 people - 12%), China (2,540 people - 7%), and Germany (2,454 - 7%), as depicted in Figure 2 - worldwide distribution.

### 4.2 Weight and meta-analytic findings

Only relationships examined three or more times in literature were considered (Santini et al., 2019), resulting in 48 types of relationships between dependent and independent variables. The Rstudio package Metafor was used for the meta-analysis. The most used dependent variables were, in descending order: behavioral intentions (19 relationships), use behavior (9 relations), satisfaction (7 relations), attitude (6 relationships), usefulness (5 relations), and ease of use (2 relationships), as indicated in Table 6. Only two of the 48 relationships did not prove to be statistically significant (p < 0.01) when assessing confidence interval, these are the relationships between behavioral intentions and privacy risk (*r* = .046) and usefulness and innovativeness of IT (*r* = .329). Behavioral intention represents the principal variable, considering the number of relationships observed, correlation, and sample. Some relations with more than ten observations are highlighted as usefulness and ease of use (14 relationships, 4,925 individuals, *r* = .643), use behavior and behavior intentions (10 relations, 4,264 respondents, *r* = .536), attitude and usefulness (13 relations, sample = 4,333, *r* = .620), and attitude and ease of use (10 relations, 4,048 individuals, r = .547), as demonstrated in Table 6.

**Table 6:** Meta and weight analysis results.

Independent Constructs	Dependent Constructs	Relations	Samples	Meta-Analysis							Weight Analysis			
				*r*	95% CI		Q	I^2^	FSN	Egger's Intercept	NonSig	Sig	Weight	Type
Usefulness	Behavioral intention	43	18,116	.559	.495	.616	1,484.09∗	97.2%	1,107,360	.837	5	38	.884	BP
Social influence		35	14,056	.410	.341	.474	776.02∗	95.6%	644,268	.440	7	28	.800	BP
Ease of use		30	13,228	.408	.324	.486	862.60∗	96.8%	435,439	.538	6	24	.800	BP
Facilitating conditions		22	8,135	.462	.460	.553	659.23∗	96.8%	181,818	.041	3	19	.864	BP
Risk		21	6,797	.292	.241	.341	100.86∗	80.2%	385,039	.863	7	14	.667	
Attitude		19	6,398	.627	.519	.715	740.59∗	97.6%	129,702	.385	0	19	1	BP
Hedonic motivation		14	7,599	.571	.403	.701	1,035.81∗	98.7%	50,642	.422	0	14	1	BP
Trust		14	4,847	.492	.372	.595	350.37∗	96.3%	74,159	.109	2	12	.857	BP
Price value		13	4,675	.271	.179	.359	133.99∗	91.0%	29,619	.072	1	12	.923	BP
Habit		12	4,624	.485	.291	.641	684.85∗	98.4%	28,934	.212	3	9	.750	
Privacy risk		11	3,627	.046	.150	.240	361.27∗	97.2%	29	.300	3	8	.727	
Self-efficacy		8	2,715	.335	.073	.553	433.91∗	98.2%	2,211	.570	3	5	.625	
Perceived behavioral control		7	1,837	.371	.194	.525	97.36∗	93.8%	18,501	.043	0	7	1	BP
Satisfaction		6	2,203	.625	.449	.755	169.74∗	97.1%	16,442	.583	0	6	1	BP
Relative advantage		5	1,194	.468	.171	.687	115.23∗	96.5%	2,833	.248	0	5	1	BP
Compatibility		5	2,253	.626	.526	.709	40.27∗	91.1%	10,641	.028	0	5	1	BP
Personal innovativeness		4	2,341	.409	.321	.491	18.36∗	83.7%	21,592	.709	0	4	1	PP
Perceived costs		4	1,880	.264	.048	.456	66.81	95.5%	4,591	.350	1	3	.750	
Perceived value		3	1,484	.735	.596	.831	36.62∗	94.5%	4,079	.074	0	3	1	PP
Behavior intentions	Use Behavior	10	4,264	.536	.417	.637	232.04∗	96.1%	67,986	.749	0	10	1	BP
Facilitating conditions		9	3,136	.321	.194	.438	116.92∗	93.2%	31,531	.294	3	6	.667	
Satisfaction		8	2,373	.582	.366	.739	335.84∗	97.9%	8,233	.025	0	8	1	BP
Habit		8	2,860	.472	.288	.622	232.46∗	97.0%	14,332	.157	0	8	1	BP
Risk		6	1,251	.319	.202	.426	24.72∗	79.8%	10,957	.331	1	5	.833	BP
Self-quarantine		6	1,627	.257	.022	.466	119.61∗	95.8%	8,392	.085	2	4	.667	
Privacy risk		5	1,989	.274	.049	.472	110.68∗	96.4%	2,932	.623	0	5	1	BP
Trust		4	1,344	.318	.023	.562	96.45∗	96.9%	1,970	.979	1	3	.750	
Usefulness		3	1,030	.472	.261	.640	30.39∗	93.4%	5,536	.291	0	3	1	PP
Confirmation	Satisfaction	8	2,586	.778	.673	.853	228.27∗	96.9%	16,900	.953	0	8	1	BP
Facilitating conditions		6	1,665	.632	.482	.746	100.47∗	95.0%	12,018	.079	2	4	.667	
Compatibility		6	1,563	.738	.624	.820	90.68∗	94.5%	6,615	.044	1	5	.833	BP
Hedonic motivation		5	1,193	.660	.626	.691	3.89	0.0%	5,079	.195	0	5	1	BP
Complexity		5	1,193	.684	.625	.736	13.20∗	69.7%	4,778	.630	1	4	.800	BP
Ease of use		4	1,332	.611	.216	.834	246.82∗	98.8%	2,847	.600	0	4	1	PP
Usefulness		4	1,495	.678	.557	.771	44.53∗	93.3%	5,100	.913	0	4	1	PP
Usefulness	Attitude	13	4,333	.620	.504	.714	376.07∗	96.7%	44,804	.144	0	13	1	BP
Ease of use		10	4,048	.547	.436	.642	164.85∗	94.5%	31,434	.117	0	10	1	BP
Hedonic motivation		5	1,535	.586	.359	.748	132.91∗	97.0%	9,628	.261	0	5	1	BP
Privacy Risk		3	973	.295	.130	.444	15.15∗	86.8%	3,505	.087	1	2	.667	
Social influence		3	1,178	.698	.496	.828	60.27∗	96.7%	3,006	.809	0	3	1	PP
Trust		3	1,221	.586	.468	.684	16.70∗	88.1%	4,080	.226	0	3	1	PP
Ease of use	Usefulness	14	4,925	.643	.637	.669	176.92∗	92.7%	63,021	.496	0	14	1	BP
Social influence		3	893	.280	.038	.491	28.61∗	93.0%	99	.224	0	3	1	PP
*Innovativeness of IT*		*3*	*2,374*	*.329*	*-.355*	*.784*	*530.59∗*	*99.6%*	*486*	*.808*	*0*	*3*	*1*	*PP*
Compatibility		3	988	.591	.135	.840	150.07∗	98.7%	1,653	.456	0	3	1	PP
Confirmation		3	1,393	.531	.212	.747	93.97∗	97.9%	1,145	.993	0	3	1	PP
Innovativeness of IT	Ease of Use	3	2,460	.392	.192	.561	54.21∗	96.3%	8,228	.673	0	3	1	PP
Trust		3	796	.661	.490	.782	26.80∗	92.5%	2,231	.844	0	3	1	PP

Note: Relations means the number of observations (our sample size); Sample represents the cumulative sum of respondents; *r* is the simple average Pearson correlation; Q is the heterogeneity result at individual and aggregate levels; CI shows the highest confidence interval; FNS is the Fail-Safe Number of articles required for the result to be false (Santini et al., 2016); Sig represents significant relationships; NonSig shows insignificant relations; BP = Best Predictor; PP = Promising Predictor; Italic line represents not supported relationship given low correlation or confidence interval difference.

**Figure 3:**
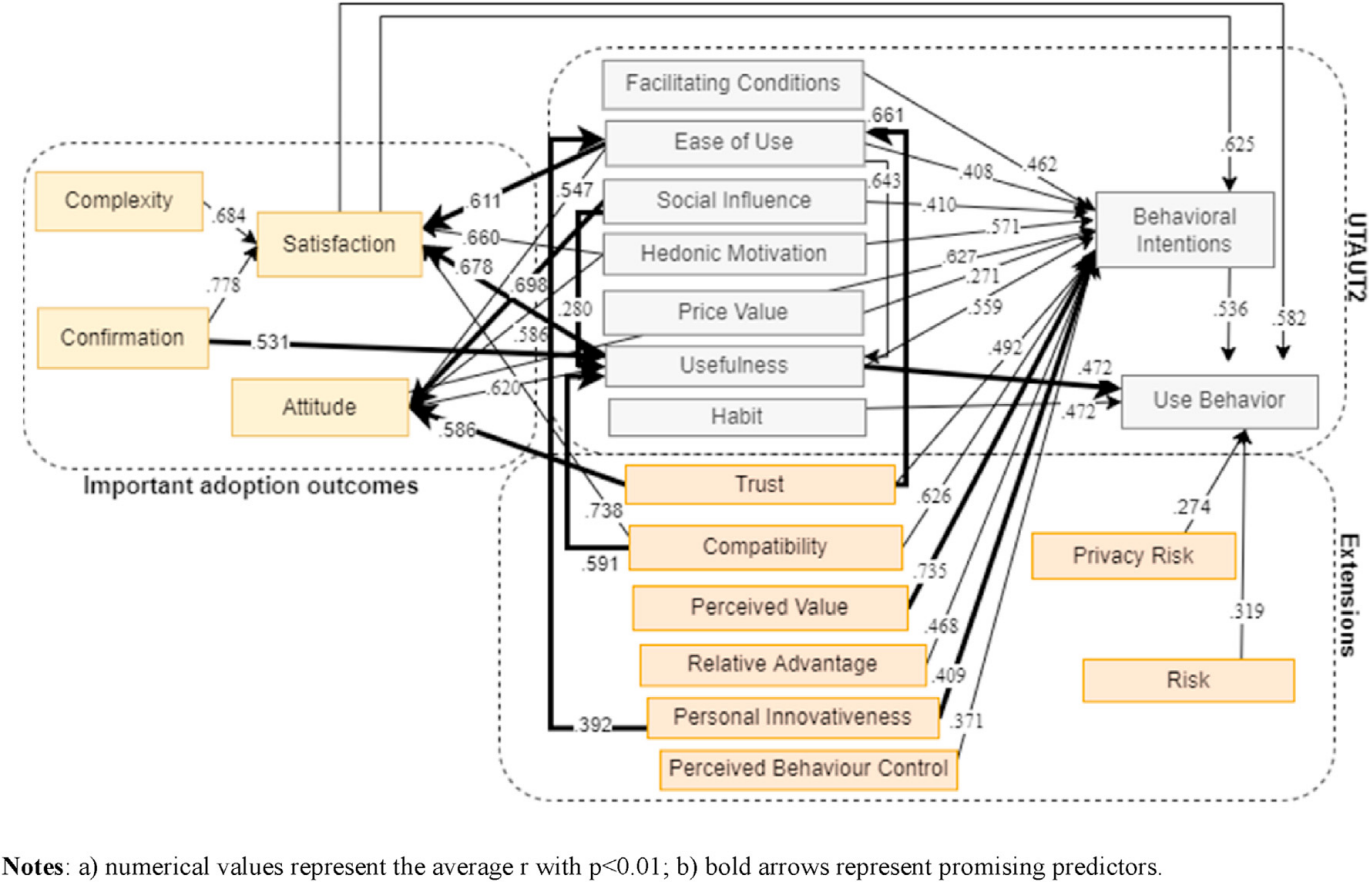
Proposed UTAUT – UTAUT2 model, with other important adoption outcomes and extensions after weight and meta-analysis.

The information presented in Table 6 denotes that attitude (*r* = .627, FSN = 129,702), satisfaction (*r* = .625, FSN = 16,442) and compatibility (*r* = .626, FSN = 10,641) are the most relevant predictors for behavioral intentions to adopt DT. For DT technologies use, behavioral intentions (*r* = .536, FSN = 67,986), satisfaction (*r* = .582, FSN = 8,233) and habit (*r* = .472, FSN = 14,332) are the key predictors. For the weight analysis, of the 48 relationships, 25 were classified as "Best Predictors" and 13 as "Promising Predictors" for individual adoption of DT, as described in Table 6. Since the UTAUT model captures the essential elements of eight other technology adoption models (Venkatesh et al., 2003), and UTAUT2 extends the original ones with new constructs (Venkatesh et al., 2012), the combination of the meta and weight analysis allowed the design of the model shown in Figure 3. It denotes the correlation (*r*) and weight among the principal variables found in this research, presenting, as in the UTAUT model, connections, possible extensions, and important outcomes for technology adoption, considering some rules like only the best and promising predictors from the weight analysis, and constructs with high correlation.

### 4.3 Moderation analysis

Considering the importance of moderators to clarify changes in the effect size of technology adoption models (Santini et al., 2019), moderation analyses were conducted at the economic, cultural, and innovation levels. Two criteria were used to select the best relations for moderation: having sufficient observations (more than 30) (Geyskens et al., 2009); and having high heterogeneity (Santini et al., 2019). Of the 48 presented relations, only three meet the criteria: behavioral intentions with the variable's usefulness (43 relations, Q = 1,484.09, I^2^ = 97.2%), ease of use (30 relations, Q = 862.60, I^2^ = 96.8%), and social influence (35 relations, Q = 776.02, I^2^ = 95.6%). As no consistent results were found among behavioral intentions and social influence, the moderation test results are only presented for the first two relations.

At an economic level, the GDP had no significant moderation effect on the relationships between behavioral intentions and usefulness (β = .599, M*low* = .553, M*high* = .506) or ease of use (β = .409, M*low* = .381, M*high* = .373). Apropos innovation indicators, positive moderation effects were found for human capital and the research dimension between behavioral intentions and ease of use (β = .336, M*low* = .438, M*high* = .309, p < 0.10), and for knowledge & technology outputs on both relations among behavioral intentions and usefulness (β = .581, M*low* = .613, M*high* = .498, p < 0.10) and ease of use (β = .363, M*low* = .453, M*high* = .334, p < 0.05). No significant moderating effect was identified for the remaining dimensions (institutions, infrastructure, market sophistication, and creative outputs). A significant moderating effect respecting the cultural context was found for individualism, on the relation among behavioral intentions and ease of use (β = .355, M*low* = .444, M*high* = .327, p < 0.05). No significant moderating effects were found for the other factors (power distance, masculinity, and uncertainty avoidance). All results are described in Table 7.

**Table 7:** Moderation analysis.

Moderator level		Behavioral intentions to usefulness			Behavioral intentions to ease of use		
		*β*	*R*	*p* value	*β*	*R*	*p* value
Sample size	Intercept			0.001			0.001
	High	1			1		
	Low						
Institutions (political, regulatory and business environment)	Intercept	0.605		0.001	0.409		0.001
	High	1	0.516		1	0.357	
	Low	0.095	0.567	0.328	0.082	0.426	0.451
Human capital and research (education, tertiary education, R&D)	Intercept	0.587		0.001	0.336		0.001
	High	1	0.500		1	0.309	
	Low	0.096	0.565	0.293	0.189	0.438	0.055∗
Infrastructure (ICTs, general infrastructure, ecological sustainability)	Intercept	0.638		0.001	0.411		0.001
	High	1	0.533		1	0.361	
	Low	0.019	0.525	0.843	0.050	0.396	0.622
Market sophistication (credit, investment, trade, competition, market scale)	Intercept	0.592		0.001	0.359		0.001
	High	1	0.501		1	0.326	
	Low	0.080	0.561	0.379	0.145	0.423	0.146
Business sophistication (knowledge workers, innovation linkages, knowledge absorption)	Intercept	0.642		0.001	0.409		0.001
	High	1	0.539		1	0.371	
	Low	0.021	0.521	0.815	0.046	0.383	0.654
Knowledge & Technology outputs (k-creation, k-impact, k-diffusion)	Intercept	0.581		0.001	0.363		0.001
	High	1	0.498		1	0.334	
	Low	0.177	0.613	0.064∗	0.206	0.453	0.038∗∗
Creative outputs (intangible assets, creative good and services, online creativity)	Intercept	0.606		0.001	0.361		0.001
	High	1	0.514		1	0.329	
	Low	0.060	0.553	0.514	0.151	0.426	0.130
Power Distance	Intercept	0.625		0.001	0.469		0.001
	High	1	0.523		1	0.401	
	Low	0.015	0.541	0.866	0.098	0.332	0.359
Individualism	Intercept	0.592		0.001	0.355		0.001
	High	1	0.510		1	0.327	
	Low	0.099	0.562	0.265	0.191	0.444	0.046∗∗
Masculinity	Intercept	0.641		0.001	0.417		0.001
	High	1	0.541		1	0.382	
	Low	0.025	0.514	0.787	0.038	0.373	0.711
Uncertainty Avoidance	Intercept	0.609		0.001	0.393		0.001
	High	1	0.518		1	0.341	
	Low	0.058	0.549	0.537	0.107	0.441	0.308
Gross domestic product	Intercept	0.599		0.001	0.409		0.001
	High	1	0.506		1	0.373	
	Low	0.063	0.553	0.486	0.045	0.381	0.655

Note: ∗∗∗p < 0.01; ∗∗p < 0.05; ∗p < 0.10.

## 5 Discussion

The global health crisis has maximized the distinct role attributed to information technology in innovative solutions for real-time communication and connectivity (Coccia, 2020). The technological advance brought about by the pandemic context accelerated service digitization and product transformation, creating the opportunity from a quantitative view for a DT update to present main factors related to individual technology adoption. By consolidating theoretical models and empirical data from previous publications, this literature review raises 442 relations important to DT between independent and dependent variables, synthesized into 48 statistically relevant relationships. To the best of our knowledge, this is the first investigation to run a meta and weight analysis correlating individual adoption and DT. The meta-analysis results show that 46 of the 48 relationships presented are statistically significant, as the relations between behavioral intention to privacy risk and usefulness to the innovativeness of IT are considered insignificant for the research. Of the six dependent constructs identified, four are included in TAM and UTAUT (usefulness, ease of use, behavioral intentions, and use behavior), indicated as most used in adoption research (Venkatesh et al., 2003). Regarding the other dependent variables, attitude emerges from TRA and TPB, while satisfaction arises from the IS success model by DeLone and McLean (1992).

Our results consolidate the validity of the primary relationships of TAM, UTAUT, and UTAUT2 models for individual adoption of DT, harmonizing apparent inconsistencies pointed out in the existing literature and bringing up a new relationship between usefulness and use behavior. The impact of social influence and facilitating conditions on DT intention and use is noteworthy. Treated as contingent variables by technology adoption theory, the presented results corroborate the importance of considering not only the technological aspects of transformation (Verhoef et al., 2021) but also the strength of social relations and peer pressure (Meske and Junglas, 2020) in addition to organizational environment and infrastructure supporting disruptive technology adoption and use (Navaridas-Nalda et al., 2020). These findings are convergent with recent bibliographic reviews on DT (Kraus et al., 2021; Mergel et al., 2019; Nadkarni and Prügl, 2021; Verhoef et al., 2021; Vial, 2019).

Moreover, the findings affirm the results of DT research applied in areas such as healthcare (Alam et al., 2020; Rodger, 2014; Yan et al., 2021), education (Navaridas-Nalda et al., 2020; Taghizadeh et al., 2021), digital services (Jain et al., 2021; Jaklič et al., 2018; Thakurta et al., 2020), blockchain (Queiroz and Fosso Wamba, 2019), Internet of Things (Aldossari and Sidorova, 2020), and artificial intelligence (Cabrera-Sá nchez et al., 2021).

Nevertheless, Scott et al. (2019) found unsatisfactory results among social influence and behavioral intentions in accessing digital services, and (Ben Arfi et al., 2021a) discovered negative effects between behavioral intention to social influence and facilitating conditions in healthcare. Usefulness and ease of use arouse particular attention since they appear as independent constructs in almost all other variables in addition to being dependent variables. It denotes the need for customization and essential characteristics like usability, utility, user experience, and ease of learning (Venkatesh et al., 2003, 2012) for DT adoption, convergent with previous studies (Baudier et al., 2020; Chopdar et al., 2018; Kamolsook et al., 2019; Khaksar et al., 2021; Pillai et al., 2020). Similar to the findings, a recent study by Aparicio et al. (2021) shows that usefulness and ease of use are determinants of the e-commerce platforms' use intentions. Contrarily, negative results were found between behavioral intentions to ease of use (Çera et al., 2020) and usefulness (Sobti, 2019).

Of the relationships presented, the top eight with the strongest correlation (*r*), all above the 0.660 limit, are (i) satisfaction with the variable's confirmation (.778), compatibility (.738), complexity (.684), usefulness (.678), and hedonic motivation (.660); (ii) behavioral intentions with perceived value (.735); (iii) attitude related to social influence (.698); and (iv) ease of use with trust (.661). Regarding the importance of satisfaction and usefulness for intentions to use, convergent results were found in the study of (Franque et al., 2021) in the mobile payment subject. Used to measure business value creation during different phases of DT (Verhoef et al., 2021), satisfaction alongside responsiveness and business scope are key strategic outcomes for DT (Cha et al., 2015). Estimated precision from the 95% confidence interval shows that some effect sizes are more accurate than others. As an example of greater precision of average effect (Borenstein et al., 2009), we cite usefulness to ease of use (.637-.669), satisfaction to hedonic motivation (.626-.691), behavioral intentions to facilitating conditions (.460-.553), and behavioral intentions to risk (.241-.341), all with narrow variation up to 100, indicating relationship robustness. Conversely, examples of less accurate estimates, with wide variations up to 700, are seen between usefulness to compatibility (.135-.840), satisfaction to ease of use (.216-.834), use behavior to trust (.023-.562), and usefulness to confirmation (.212-.747), denoting weak connections (Borenstein et al., 2009).

Relevant results were found for cultural moderators, as high individualism strengthens the relationship between ease of use and behavioral intentions. A digital culture driven by DT can be the key to these findings, empowering the individual as an autonomous body responsible for building their own will (Guy, 2019). Individualistic people will strive harder to learn new technologies (Blut et al., 2021) as they think more about themselves as a path to self-fulfillment and personal success, demonstrating social competence according to high standards of reference (Veiga et al., 2001). Otherwise, collectivist cultures do not have a reference for success, devaluing the individual fluency of IT to keep bonds and prestige with colleagues, leading individuals who are more intellectually equipped to hide their knowledge (Veiga et al., 2001). Given that DT is linked to introducing new technology, bringing changes and resistance in collectivist cultures, it favors the individualistic context with the need for better usability (Harrati et al., 2016; Hofstede, 2001). No moderating effect was found in the tested relations for the remaining cultural factors such as power distance, masculinity, and uncertainty avoidance. Recognizing that our results may have been influenced by the large volume of studies about mobile applications for DT, the findings are convergent with Blut et al. (2021), as the effects of usefulness and ease of use on behavioral intentions and use are more substantial for mobile users.

The most significant moderation result came from innovation indicators. Knowledge and technology outputs represent intellectual capital and strengthen both relationships of behavioral intentions (usefulness and ease of use), comprising as sub-pillars knowledge creation, the impact of innovations at the micro and macroeconomic level, and knowledge absorption (WIPO, 2020). The results show that countries with high intellectual capital scores have higher readiness levels for DT and system adoption (Švarc et al., 2020). The knowledge-free environment brought by intellectual capital provides innovation and raises the standard for creating new products (Švarc et al., 2020), being responsible for DT market expansion, faced by the pressure for better usability and utility required by high-quality applications (Ståhle et al., 2015). Reflecting the pattern of education and innovation in some economies, human capital and research impact the relation among intentions and ease of use, being composed by the quality of R&D activities, elementary, secondary, and higher education (WIPO, 2020). Contexts with immense human capital reflect ample quality education, demanding high standards for disruptive technology solutions, such as the quality of personal skills and professional training positively impacts national readiness for DT (Švarc et al., 2020). Well-educated individuals strengthen the relationship between ease of use and behavioral intentions in the context of technology adoption (Baudier et al., 2020; Sobti, 2019). No relevant moderation results were seen for the remaining innovation indicators, as Table 7 exemplifies. Moderation by GDP had no relevant effect, similarly to (Franque et al., 2020). However, the importance of this moderator for DT adoption suggests that further studies are imperative to consider that countries with high economic development tend to have better technology adoption (Kim and Peterson, 2017).

A major finding of this research is to have satisfaction as the most relevant outcome for DT, because in addition to being the construct that has the highest correlations, it is the only dependent variable in which the correlation (β) is greater than .610 in all significant relationships with the independent variables, which are: confirmation (0.778), compatibility (0.738), complexity (0.684), usefulness (0.678), hedonic motivation (0.660), facilitating conditions (0.632), and ease of use (0.611).

**Figure 4:**
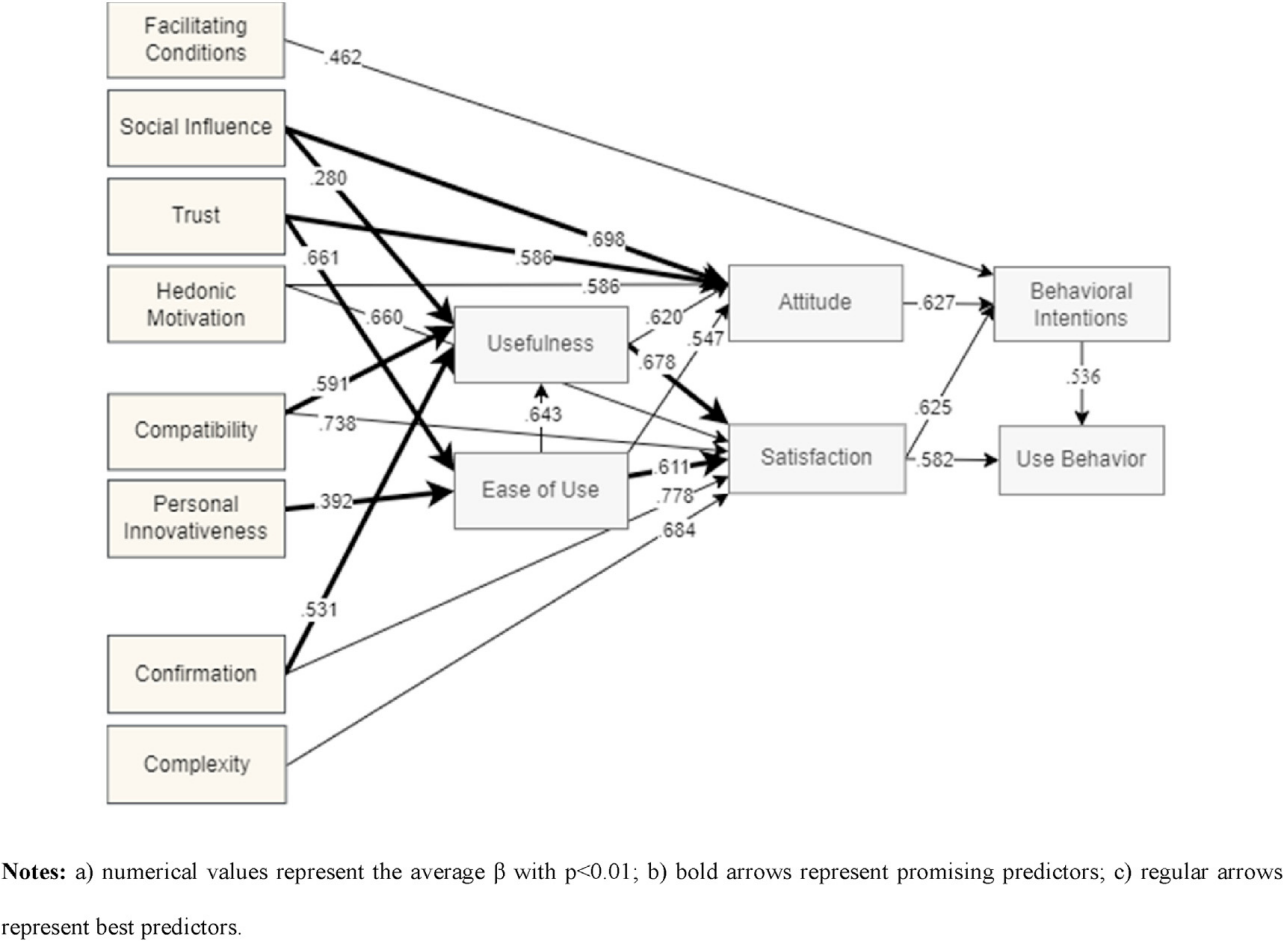
Proposed model considering different outcomes of UTAUT as results of weight and meta-analysis.

The result of weight and meta-analysis made it possible to assess the statistical significance and select the central relationships between variables (Blut et al., 2021), indicating the most relevant factors for the adoption of DT and making it possible to see the predictive power of the variables satisfaction and attitude, not considered in the two main models of TAM and UTAUT. In this context, it was possible to propose a new theoretical model capable of benefiting future research on DT, focusing on different outcomes of intention and use of technology, as shown in Figure 4. The construction of the presented model followed the criteria: (i) statistically significant relationships through the meta-analysis, and (ii) only relationships classified as best and promising predictors, according to weight analysis.

The model brings satisfaction and attitude as primary outcomes, also considering the relevance of two other critical variables for adopting DT, common to the TAM and UTAUT models (Venkatesh et al., 2003), which are usefulness and ease of use. All variables included have a broad theoretical basis, proven by the large number of studies that use them (Venkatesh et al., 2012), providing reliability and solidity to the use of the model in future research on DT.

Even not being considered the best predictor by weight analysis, given the lack of only one statistically relevant relationship, the facilitating conditions construct was kept in the model due to its great importance for DT. Representing the company's support and resources (Venkatesh et al., 2003), available for the adoption of DT, facilitating conditions brings the contingent factor of culture and organizational context to the complex equation (Vial, 2019), expanding the scarce debates about the ideal structure for the success of DT strategies (Verhoef et al., 2021).

### 5.1 Impacts on research and practice

This weight and meta-analysis synthesize the most relevant previous publications on DT individual adoption, drawing an overview of the subject and presenting new practical and theoretical ideas, advancing the research area. For theoretical advances, predictors of DT are clarified, following technology adoption theory, to present a UTAUT model extension, with new consolidated constructs and different outcomes for future investigations, such as satisfaction and attitude (Blut et al., 2021).

Convergent to the weight and meta-analysis conducted by Franque et al. (2020), amidst the most used factors, our results present a new UTAUT 2 model with extensions proper to the DT context, as shown in Figure 3. We also suggest, more broadly, a comprehensive model as Figure 4 indicates, which considers best and promising predictors, by mixing a comprehensive variety of constructs, from different technology adoption theories, capable of integrating the context of disruptive technologies, significantly impacting the individual adoption of DT technologies, namely the technology acceptance model (Davis, 1989), IS success model (DeLone and McLean, 1992), expectation confirmation model (Bhattacherjee, 2001), theory of planned behaviour (Ajzen, 2011), and the unified theory of acceptance and use of technology in both versions (Venkatesh et al., 2003, 2012).

With the proposition of an extended model for technology adoption of DT with an individual focus, we bring new constructs and elements to improve the research field. The model expansion shows the importance of a holistic view for managerial advances (Orsingher et al., 2016), highlighting the need for contextual and social analysis to the already existing complexity of disruptive technology ground (Selander and Jarvenpaa, 2016). DT practices involve introducing digital solutions that require changes in the way people work, changing organizational processes and roles to business model disruption (Kraus et al., 2021). For practical implications, innovative technology needs to be compatible with individual and organizational problems, considering the values and experiences of adopters (Selander and Jarvenpaa, 2016). The importance of individual satisfaction as an outcome reflects the success of DT, measured by consumers, customers, citizens, and employees as technology adopters. Network of individuals' relationships and organizational context provide essential resources and infrastructure to disruptive technology adoption. Managers need to consider technology compatibility for users or organizations adopting new technologies since it brings the challenge of supplanting an already known ecosystem through changes (Blut et al., 2021).

The impact of the constructs risk and privacy risk shows the importance of information management for individuals and the need to minimize risks through management to ensure that rules are followed, reducing possible barriers for the transformative initiatives (Nadkarni and Prügl, 2021). The importance of attitude, intention, and use of disruptive technologies, and the need for investments in user-client experience was also revealed, as friendly platforms should offer useful and relevant information, be attractive, intuitive, and easy to use, increasing customer interaction, satisfaction, and performance (Gupta et al., 2020; Harrati et al., 2016). Managers should be aware of the factors related to digital technology adoption by consumers and customers, such as fun or pleasure, habit strength, transaction costs, and the relations among cost/benefit and time/effort. Blut et al. (2021) identified habit as the standout predictor among the original ones and stated personal innovativeness as a key characteristic for technology adoption decisions.

Considering the model results shown in Figure 4, we can state the importance of variables such as satisfaction and attitude impacting behavioral intentions and usage behavior for DT, also acting as mediators of other variables. In this sense, to encourage the use of DT tools, managers should focus on user attitude, satisfaction, and the precursor factors. For satisfaction, it is crucial to consider social influence from family and close friends, user confidence, fun or pleasure, ease of use, and all the benefits for users. For satisfaction, managers can be aware of the perception of difficulty in understanding and using some innovation, ease of use, usefulness, validation of the individual's basic expectations after interaction, fun, and pleasure, and tool compatibility. The perception of support and available resources is a precursor of behavioral intentions for using the DT tool.

The moderation analysis reflects that individualism, as a cultural factor, human capital and research, and knowledge & technology output, as innovative factors, amplify the strength of the relationship between behavioral intentions to usefulness and ease of use for individual DT adoption. Managers should invest in employees' capabilities and autonomy by promoting IT champions (Harrati et al., 2016), as group management can be less important than managing individual needs (Hofstede, 2001). Given the findings in the innovation context, the moderation role of human capital and research highlights the importance of employee training and development investments (Švarc et al., 2020). The indicator knowledge & technology innovation output denotes the need for an ideal environment for constant diffusion and exchange of knowledge among individuals (Guggemos and Seufert, 2021; Pillet and Carillo, 2016), strengthening ease of use and usefulness perceptions to adopt DT technologies.

### 5.2 Limitations and future research

This work has several limitations. First, not all studies on DT adoption were included since many presented different statistical methods, did not contain sufficient quantitative data, or were not presented in the English language, including lacunae in sample data and statistical correlation values. Research scope expansion allows the inclusion of publications with diverse statistical methods to new analysis with comprehensive results. Second, additional attention should be given to the magnitude of DT's risk and privacy risk variables, even after non-statistically significant results, requiring additional research due to the considerable value of their relationship with use behavior and attitude. Vimalkumar et al. (2021) stated that privacy risk is mediated by privacy concerns and trust for disruptive technology adoption. Third, most publications do not have information about survey items, making it impossible to identify certain similarities among variables and limiting the coding and merging process. In this research, not all variables with similar nomenclature present similarity of meaning, as illustrated in Table 1. Fourth, few moderating dimensions were considered, bringing economic, cultural, and innovation contexts. Culture is an essential factor in technology adoption (Srite and Karahanna, 2006), and future studies should consider not only four dimensions (Hofstede, 2001) but all others (Blut et al., 2021). Including new relevant moderators for DT individual adoption can be relevant as user characteristics (Blut et al., 2021), technology, or sample type (Santini et al., 2019). Finally, as DT adoption can be understood from an individual or organizational view, we recommend carrying out a meta-analysis about firms' views to provide a complete perception by comparing individual and firms' adoption factors.

## 6 Conclusions

DT has gained strength and importance in academic and practical contexts, impacting people's lives, given the acceleration in the adoption of disruptive technologies brought about by the pandemic implications. To understand this context, a weight and meta-analysis was carried out to synthesize and aggregate previous literature to advance the theme, suggesting new constructs and relations for further investigation. After a literature review, 88 publications and 99 datasets were found, comprising 442 relevant relationships, considering those examined at least three times in the literature. The weight and meta-analysis made it possible to analyze relations, clarifying 46 statistically significant relationships out of 48. Constructs of great impact in individual adoption literature were identified and presented through a theoretical model that extends existing academic research and innovates, proposing new outcomes for disruptive technology adoption. For Blut et al. (2021), meta-analysis can lead to the precise specification of some theory applied to a context, given the variability of user type, national cultural aspects, and technologies applied in previous research. This weight and meta-analysis contributed to overcoming conflicting results from the primary studies presented. For example, we showed the existence of a positive and moderate relationship between ease of use and behavioral intentions, convergent with Khaksar et al. (2021) and contrary to Vimalkumar et al. (2021), which showed a negative relationship. Another positive and moderate relationship found by us was among usefulness and behavioral intentions, similar to Kabir (2020) and contrary, as shown by Nastjuk et al. (2020).

As significant contributions for the DT field of study, our results highlighted that attitude and satisfaction are relevant predictors of behavioral intentions and are promising outcomes for further investigation, including compatibility and personal innovativeness. Behavioral intentions, satisfaction, and habit are the best predictors for disruptive technology use. Usefulness and ease of use play a critical role in DT, influencing outcomes like intention, use, satisfaction, and attitude, moderated by cultural and innovative contexts. Individualism, representing the cultural aspect and the indicators of human capital and research, and knowledge and technology output, as the innovative element, moderate the relationship between behavioral intentions to usefulness and ease of use. Contributions made after presenting the results provide an update on DT's state of the art combined with individual technology adoption, bringing advantages for future research.

## Declarations

### Author contribution statement

Diego Cavalcanti: Conceived and designed the experiments; Performed the experiments; Analyzed and interpreted the data; Contributed reagents, materials, analysis tools or data; Wrote the paper.

Tiago Oliveira: Conceived and designed the experiments; Contributed reagents, materials, analysis tools or data; Wrote the paper.

Fernando de Oliveira Santini: Analyzed and interpreted the data; Wrote the paper.

### Funding statement

This work was supported by national funds through 10.13039/501100001871FCT (Fundação para a Ciência e a Tecnologia) under the project - UIDB/04152/2020 - Centro de Investigação em Gestão de Informação (MagIC).

### Data availability statement

No data was used for the research described in the article.

### Declaration of interests statement

The authors declare no conflict of interest.

### Additional information

No additional information is available for this paper.
